# Global Autism Spectrum Disorder Prevalence Estimates and Associated Covariates: A Systematic Review and Meta-Regression Analysis

**DOI:** 10.7759/cureus.106260

**Published:** 2026-04-01

**Authors:** John K Muthuka, Chrisphine Onyango, Japheth M Nzioki, Lucy K Chebungei, Ruvimbo Zimunya, Andrina Simengwa, Sara Kim, Desire A Nshimirimana, Rosemary Nabaweesi

**Affiliations:** 1 Community Health and Health Promotion, Kenya Medical Training College, Nairobi, KEN; 2 School of Global Health, Meharry Medical College, Nashville, USA; 3 Children Behavior Services, Anderson Center for Autism, New York, USA; 4 School of Nursing, Andrews University, Berrien Springs, USA; 5 Department of Nursing, Kenya Medical Training College, Nairobi, KEN; 6 College of Doctoral Studies, Grand Canyon University, Phoenix, USA

**Keywords:** autism spectrum disorder (asd), bayesian hierarchical modeling, global epidemiology, global mental health, prevalence, risk predictors, systematic review and metaanalysis

## Abstract

This review aimed to estimate the global prevalence of autism spectrum disorder (ASD) and identify methodological and contextual covariates influencing prevalence estimates. Despite increasing research, the true global burden of ASD remains unclear due to substantial variability in reported prevalence across studies. The specific methodological and contextual factors driving this heterogeneity have not been fully quantified. Electronic databases, including PubMed, Scopus, Web of Science, Embase, and Google Scholar, were systematically searched from 2004 to 2025. All human population-based studies were included, irrespective of region or diagnostic framework. Nineteen studies, comprising approximately 20.6 million participants, met the inclusion criteria and reported ASD prevalence. To estimate pooled prevalence, a random-effects model (REML) was used, and Bayesian hierarchical modeling was conducted to provide posterior estimates. Publication bias and sensitivity analyses were conducted, followed by meta-regression to account for covariates such as diagnostic framework, study setting, and region. The frequentist pooled prevalence of ASD was 0.8% (95% CI: 0.4%-1.7%), with substantial heterogeneity (I² ≈ 100%) and a wide prediction interval (0.03%-17.3%). Bayesian hierarchical modeling produced a posterior mean prevalence of 1.55% (95% CrI: 0.75%-4.1%), and a Bayesian sensitivity analysis excluding an extreme outlier study yielded a posterior mean prevalence of 0.9% (95% CrI: 0.6%-1.8%) with substantial heterogeneity (τ = 1.14, I² = 99.98%) and a prediction interval of 0.09%-9.5%, indicating strong consistency between frequentist and Bayesian estimates. Regional prevalence varied: North America (1.9%), Africa (6.3%) (wide confidence intervals reflecting limited data), Europe (0.4%), Latin America (0.2%), and the Middle East (0.6%). Meta-regression analysis demonstrated that ASD prevalence was influenced by diagnostic framework, study setting, and region (R² ≈ 0.78), and the combined impact of all covariates explained a substantial proportion of the variance in effect sizes. Our updated meta-analysis indicated that global ASD prevalence varies widely across regions, largely due to methodological and contextual differences rather than demographics alone. Standardized diagnostic practices, enhanced surveillance, and targeted investment in underrepresented regions are critical for improving the accuracy and comparability of prevalence estimates. This updated synthesis provides a more robust and context-sensitive estimate of global ASD prevalence and supports the interpretation of variability observed in prior studies.

## Introduction and background

Autism spectrum disorder (ASD) is a neurodevelopmental condition characterized by persistent differences in social communication and restricted, repetitive patterns of behavior that emerge in early childhood [1,2]. The global burden of ASD has become an increasing public health priority due to its lifelong functional implications and substantial social and economic costs. Accurate prevalence estimates of ASD are crucial for informing health policy, allocating resources, and guiding early detection and intervention strategies [3].

In recent decades, the reported prevalence of ASD has risen considerably across various regions. Meta-analytic studies suggest that global prevalence estimates generally range from 0.6% to 1.0%, but there is substantial variation across continents and study designs [4]. The Global Burden of Disease (GBD) Study [5,6] further underscores marked geographic disparities in prevalence and associated disability burden. Despite growing global surveillance efforts, significant variability in prevalence estimates remains, raising methodological and interpretative concerns that demand attention.

Much of this variation may stem from differences in case ascertainment methods, diagnostic frameworks (such as Diagnostic and Statistical Manual of Mental Disorders, 4th Edition (DSM-IV), Diagnostic and Statistical Manual of Mental Disorders, 5th Edition (DSM-5), International Classification of Diseases, 10th Revision (ICD-10), and ICD-11), sampling strategies, age composition, and healthcare infrastructure, rather than reflecting true epidemiological differences. Indeed, diagnostic criteria for ASD have evolved substantially across DSM and ICD revisions, leading to changes in case definitions that could contribute to non-equivalent prevalence estimates across time and settings [1,2].

While previous meta-analyses have predominantly focused on pooled prevalence estimates or regional summaries, there has been comparatively limited attention given to systematically quantifying the study-level drivers of between-study heterogeneity. Given the ongoing changes in diagnostic practices, awareness, and surveillance capacity from 2004 to 2025, an updated synthesis that explicitly examines sources of heterogeneity is needed. The primary objective of this study was therefore twofold: first, to estimate pooled global and regional ASD prevalence; and second and most important, to identify and quantify the methodological and contextual moderators contributing to the observed heterogeneity in reported prevalence estimates using multivariable meta-regression techniques.

## Review

Materials and methods

Design, Protocol, and Registration

This systematic review and meta-analysis followed established guidelines for evidence synthesis of observational prevalence studies. Reporting adhered to the Preferred Reporting Items for Systematic Reviews and Meta-Analyses (PRISMA) 2020 guidelines [7]. The current review is part of a larger, prospectively registered study titled Global Trends in Autistic Disorder Diagnosis and Intervention Outcomes: A Systematic Review, Meta-analysis, and Meta-Regression (International Prospective Register of Systematic Reviews (PROSPERO): CRD420251069271), with a particular emphasis on regional and socioeconomic differences.

Selection Criteria and Search Strategy

Our systematic review included observational studies published in English that reported ASD prevalence between 2004 and 2025. Eligible articles used standardized diagnostic frameworks (DSM-IV, DSM-5, ICD-10, or ICD-11), with ICD-10 diagnoses such as childhood autism, atypical autism, and Asperger syndrome considered ASD-equivalent. They were eligible if they were population- or institution-based (hospital- or clinic-based) and reported ASD prevalence. Those based on general population samples, including school- and community-based populations, were included provided they offered extractable prevalence data. We excluded non-ASD studies, duplicate datasets, non-English publications, and article types such as case reports, case series, reviews, meta-analyses, editorials, or commentaries. Studies lacking full text, clear diagnostic criteria, or extractable prevalence data were also excluded, as were genetic, neurobiological, or laboratory-based studies without population-level estimates. A comprehensive search was conducted in PubMed/MEDLINE, Embase, Web of Science, and Scopus for studies published between January 1, 2004, and December 31, 2025, along with grey literature sources. The strategy combined controlled vocabulary (MeSH terms) and free-text keywords such as: ("autism spectrum disorder"[MeSH] OR "autism spectrum disorder" OR "ASD" OR "autism") AND ("Prevalence"[MeSH] OR prevalence OR "epidemiology" OR "occurrence" OR "frequency") AND ("Epidemiologic Studies"[MeSH] OR "cross-sectional studies" OR "population-based" OR "survey"). No geographic restrictions were applied.

Study Selection

Two independent reviewers (JM and CO) screened all records at the title and abstract level. Eligible studies underwent full-text review by two additional independent reviewers (LC and JM). Discrepancies were resolved by consensus, with arbitration by a senior reviewer (RN) if needed. 

Data Extraction

Two reviewers independently extracted data using a standardized form. Variables extracted included author, year of publication, region, diagnostic framework, age group, sample size, study design, sampling method, study setting, numerator and denominator for ASD prevalence, sex-specific estimates, and relevant contextual characteristics. Prevalence values were transformed to proportions for analysis, and confidence intervals were recalculated where necessary using binomial methods. Studies lacking clear diagnostic criteria or denominators were excluded from quantitative synthesis. Studies were further classified as population-based or institution-based according to their sampling setting.

Outcomes

The outcome of interest was the prevalence of autism spectrum disorder (ASD), defined as cases identified in the included studies using standardized diagnostic criteria (e.g., DSM-IV, DSM-5, ICD-10, ICD-11) or validated assessment tools. Differences in case definitions and diagnostic methods across studies were documented and considered in the interpretation of results. Secondary variables examined for moderator analyses included sex-specific prevalence, mean or median age, geographic region, diagnostic framework, and study year.

Risk of Bias and Quality Assessment

Risk of bias was assessed using the Newcastle-Ottawa Scale (NOS) [8] for observational studies. This tool evaluates studies across three domains: selection of study groups, comparability of groups, and ascertainment of the outcome of interest, with a maximum score of nine stars. Studies scoring 7-9 stars were considered low risk of bias (high quality), 4-6 stars as moderate risk of bias (moderate quality), and 0-3 stars as high risk of bias (low quality). Only studies classified as low or moderate risk of bias were included in the systematic review, while studies with high risk of bias were excluded. Disagreements in scoring were resolved through consensus between reviewers.

Statistical Analyses

Meta-analyses were performed using Jeffrey’s Amazing Statistics Program (JASP) (Version 0.95.4.0, University of Amsterdam, Amsterdam, Netherlands) [9] with random-effects models estimated via restricted maximum likelihood (REML). Prevalence estimates were logit-transformed to stabilize variance and subsequently back-transformed for interpretability. Between-study heterogeneity was assessed using Cochran’s Q statistic, I², and τ², while influence diagnostics and Baujat plots were used to identify studies exerting disproportionate effects on pooled estimates. Funnel plots were generated to evaluate potential small-study effects, with asymmetry interpreted cautiously, and residual funnel plots adjusted for study-level moderators were additionally examined. Meta-regression analyses explored pre-specified moderators, including geographic region, diagnostic framework, mean age, study setting, and study year. Univariable analyses were conducted initially, followed by a reduced multivariable model including statistically significant moderators. Multicollinearity was assessed using variance inflation factors (VIF), and model stability was evaluated through leave-one-out diagnostics and influence analyses. Sensitivity analyses included leave-one-out procedures, exclusion of statistical outliers, exclusion of studies using earlier diagnostic frameworks (DSM-IV/ICD-10), removal of poor-quality studies, and exclusion of studies with unspecified diagnostic frameworks to assess the robustness of pooled prevalence and moderator effects. Additionally, Bayesian random-effects meta-analysis was conducted using weakly informative priors, specifying a Normal (0,1) prior for pooled logit prevalence and a Half-Cauchy (0,1) prior for between-study heterogeneity (τ). Markov Chain Monte Carlo sampling was performed with appropriate burn-in and iterations, and convergence was confirmed using R-hat statistics and effective sample sizes.

Results

Included Articles and Quality Assessment (Systematic Review)

**Figure 1:**
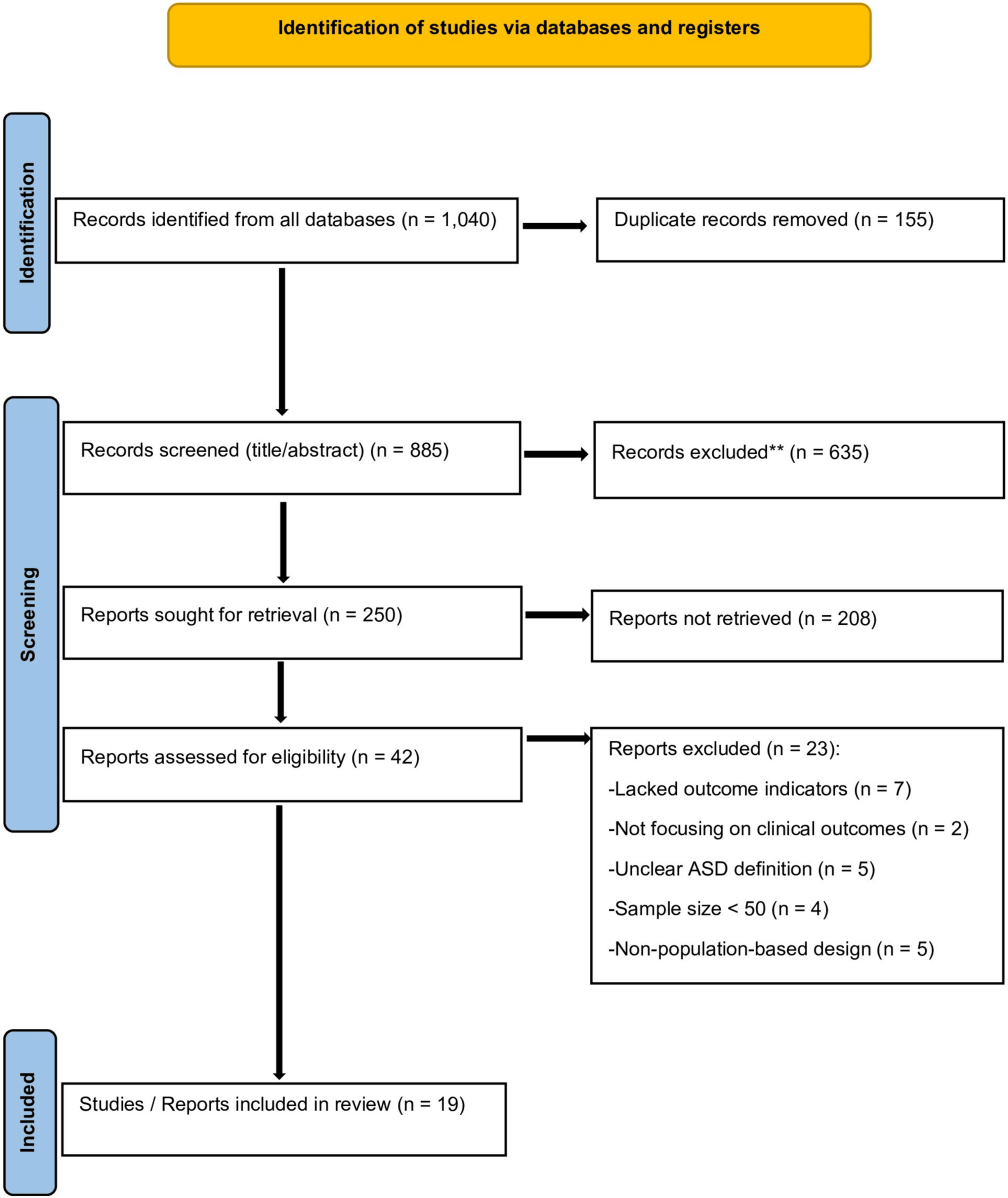
Identification and selection of studies/records in the databases ASD: autism spectrum disorder. PRISMA: Preferred Reporting Items for Systematic Review and Meta-Analysis. **Records excluded after title/abstract screening due to not meeting inclusion criteria (wrong population, intervention, outcome, or study design). Study Setting: Included studies comprised both population-based and institution-based designs as per eligibility criteria.

A total of 1,040 records were retrieved from four databases: PubMed/MEDLINE (n = 415), Embase (n = 234), Web of Science (n = 257), and Scopus (n = 134). All records were imported into Rayyan for duplicate removal and screening. After removing 155 duplicates, 885 records remained for title and abstract screening. Of these, 635 were excluded, and 250 reports were sought for retrieval. A total of 208 reports could not be retrieved, leaving 42 full-text articles assessed for eligibility. After full-text evaluation, 23 articles were excluded for predefined reasons. Ultimately, 19 studies [10-28] met the inclusion criteria and were included in the quantitative synthesis (Figure 1). The full search strategies and database-specific counts including exclusion reasons are provided in Supplementary materials* *1, 2*.*

Features of the Included Studies

The 19 included studies (n = 20,624,959) evaluated the prevalence of ASD across diverse geographic regions and study settings. Considerable heterogeneity was observed in study design, population characteristics, and diagnostic approaches. Six studies employed survey- or community-based methodologies conducted at national or community levels [10,11,13,19,20,23]. One additional community-based study conducted in Egypt also met these criteria [28]. Four studies utilized registry-based data sources for prevalence estimation [16-18,25]. Surveillance-based monitoring systems were used in three studies [14,22,25], while three investigations were conducted in school-based populations [15,24,26]. One study was conducted in a tertiary clinic-based setting and one study used claims-based clinical records to estimate prevalence [27]. Diagnostic frameworks across studies primarily included DSM-IV, DSM-5, and ICD-10 criteria. Reported ASD prevalence ranged from 0.09% in a school-based European study [24] to 2.79% in a claims-based population study [27]. An extreme prevalence estimate of 45% was reported in a clinic-based tertiary hospital study [12] which likely reflects referral bias and methodological differences rather than population prevalence (Table 1).

**Table 1:** Features of the studies included in the meta-analysis (n = 19) ASD: autism spectrum disorder; DSM-IV: Diagnostic and Statistical Manual of Mental Disorders, 4th Edition; DSM-5: Diagnostic and Statistical Manual of Mental Disorders, 5th Edition; ICD-10: International Classification of Diseases, 10th Revision; CASI: Childhood Autism Spectrum Inventory; ADDM: Autism and Developmental Disabilities Monitoring. Christensen et al., 2019 [14]: Sample size (N): 58,467-70,887 (age 4); 56,727-71,928 (age 8). Jensen et al., 2014 [18]: Approximate prevalence we estimated: 0.38%; May et al., 2017 [23]: The overall combined ASD prevalence estimated at 2.25%, corresponding to ≈225 cases in 10,000 children, based on overlapping parent- and teacher-reported cases.

Study/Author	Location	Study Design and Setting	Diagnostic Framework	Year(s)	Total Sample Size (n)	ASD Cases (n)	Prevalence (%)	Study Type
Al-Mamari et al. [10]	Middle East	Cross-sectional, National Survey	DSM-IV, ICD-10	2017-2018	835,784	1,705	0.20	Survey-based
Alshaban et al. [11]	Middle East	Cross-sectional, National Sample	DSM-IV, ICD-10	2015-2018	176,960	1,410	0.80	Survey-based
Arinda et al. [12]	Africa	Clinic-based, Tertiary Hospital	DSM-IV	2019-2020	318	143	45.0	Clinic-based
Arun and Chavan [13]	South Asia	Community-based, Stratified	DSM-IV, CASI	2019	8,451	19	0.22	Community-based
Christensen et al. [14]	North America	Surveillance-based, 7 Sites	DSM-IV, DSM-5	2010-2014	70,587	1,085	1.50	Surveillance-based
Dekkers et al. [15]	Latin America	School-based, Urban School	DSM-IV	2015	51,453	165	0.32	School-based
García-Zambrano et al. [16]	Latin America	Registry-based, National	ICD-10	2018	9,980,000	18,695	0.19	Registry-based
Hinkka-Yli-Salomäki et al. [17]	Europe	Registry-based, Hospital	ICD-10	1987-2005	1,200,000	6,444	0.54	Registry-based
Jensen et al. [18]	Europe	Registry-based, Psychiatric	ICD-10	1995-2010	3,947,000	14,997	0.38	Registry-based
Kamal et al. [19]	Middle East	Survey-based, National Survey	DSM-IV, ICD-10	2015-2018	1,493	17	1.14	Survey-based
Kogan et al. [20]	North America	Survey-based, National Survey	DSM-IV, DSM-5	2016	1,500,000	37,500	2.50	Survey-based
Kouznetsov et al. [21]	Europe	Registry-based, National	ICD-10	2021	1,666,014	15,706	0.94	Registry-based
Maenner et al. [22]	North America	Surveillance-based, ADDM	DSM-5	2020	564,000	5,643	1.00	Surveillance-based
May et al. [23]	Oceania	Survey-based, National Cohort	DSM-IV	2006-2016	10,000	225	2.25	Survey-based
Oliveira et al. [24]	Europe	School-based, School	ICD-10	2007	343,718	322	0.09	School-based
Redfield et al. [25]	North America	Surveillance-based, ADDM	DSM-IV	2014	219,913	5,058	2.30	Surveillance-based
Skonieczna-Żydecka et al. [26]	Europe	School-based, School	ICD-10	2010-2014	2,514	9	0.35	School-based
Xu et al. [27]	North America	Claims-based, Clinical Records	ICD-10, DSM-5	2016	43,032	1,229	2.79	Claims-based
Yousef et al. [28]	Africa	Community-based, Screening	DSM-IV	2019	3,722	20	0.54	Community-based

Risk of Bias in Included Studies

Risk of bias was assessed using an adapted Newcastle-Ottawa Scale for cross-sectional and prevalence studies [29]. Nine studies were judged to have a low risk of bias, exhibiting comprehensive population coverage, standardized diagnostic criteria, and robust outcome measures [14,16-18,20-22,25,27]. Seven studies showed a moderate risk of bias, primarily due to selection bias and limitations in outcome ascertainment, particularly in survey-, school-, and community-based studies [10,11,13,15,23,24,26]. Three studies showed a high risk of bias due to limited representativeness and potential outcome misclassification in clinic-based, screening-based, and modeled-estimate studies [12,19,28] (Table 2).

**Table 2:** Risk of bias assessment of included studies using the adapted Newcastle-Ottawa Scale Adapted Newcastle-Ottawa Scale [29].

Study	Selection (5)	Comparability (2)	Outcome (3)	Total (10)	Risk of Bias
Al-Mamari et al. [10]	3	1	2	6	Moderate
Alshaban et al. [11]	3	1	2	6	Moderate
Arinda et al. [12]	2	0	2	4	High
Arun and Chavan [13]	3	1	2	6	Moderate
Christensen et al. [14]	5	2	3	10	Low
Dekkers et al. [15]	3	1	2	6	Moderate
García-Zambrano et al. [16]	5	2	3	10	Low
Hinkka-Yli-Salomäki et al. [17]	5	2	3	10	Low
Jensen et al. [18]	5	2	3	10	Low
Kamal et al. [19]	2	0	2	4	High
Kogan et al. [20]	4	2	2	8	Low
Kouznetsov et al. [21]	5	2	3	10	Low
Maenner et al. [22]	5	2	3	10	Low
May et al. [23]	3	1	2	6	Moderate
Oliveira et al. [24]	3	1	2	6	Moderate
Redfield et al. [25]	5	2	3	10	Low
Skonieczna-Żydecka et al. [26]	3	1	1	5	Moderate
Xu et al. [27]	4	2	2	8	Low
Yousef et al. [28]	2	0	1	3	High

Meta-Analysis of the Pooled Prevalence Estimate of Autism Spectrum Disorder (ASD) 

**Figure 2:**
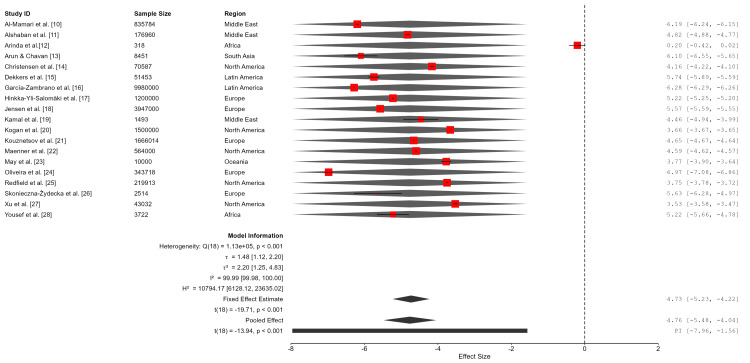
Forest plot of the pooled prevalence of autism spectrum disorder (ASD) across studies

A random-effects meta-analysis was conducted to estimate the pooled prevalence estimate of ASD. Values were calculated on the logit-transformed scale, and the estimated prevalence was 0.8% (95% CI: 0.4%-1.7%). Between-study heterogeneity was extremely high (Q (18) = 1.13 × 10⁵, p < 0.001; I² = 99.99%; τ² = 2.20), indicating that nearly all observed variability reflected true differences across study populations rather than sampling error. The 95% prediction interval ranged from 0.03% to 17.3%, suggesting that ASD prevalence in a new population could vary substantially depending on regional, methodological, and diagnostic factors. As such, the pooled estimate should be interpreted as an average across highly diverse contexts rather than as a single globally representative prevalence rate (Table 3; Figure 2).

**Table 3:** Pooled prevalence estimate of autism spectrum disorder and heterogeneity statistics The pooled effect is transformed using log odds to proportions transformation. Pooled prevalence and 95% confidence interval (CI) represent the average effect across studies. The 95% prediction interval (PI) indicates the range in which the prevalence of ASD is expected to fall in a new population. Heterogeneity statistics reflect the degree of variability across studies beyond chance. ASD: autism spectrum disorder.

Statistic	Estimate (95% CI/PI)
Pooled prevalence (%)	0.8 (0.4-1.7)
95% Prediction interval (%)	0.03-17.3
Heterogeneity	Qₑ = 1.13 × 10⁵, p < 0.001; I² = 99.99%; τ² = 2.20 (1.25-4.83); τ = 1.48 (1.12-2.20)

Model fit statistics indicated comparable results for maximum likelihood (ML) and restricted maximum likelihood (REML) estimation. The REML model demonstrated slightly better fit (log-likelihood = -32.70; Akaike information criterion (AIC) = 69.40; Bayesian information criterion (BIC) = 71.18; corrected Akaike information criterion (AICc) = 70.20) and was therefore retained for inference (Table 4).

**Table 4:** Fit measures for the random-effects meta-analysis model ML: maximum likelihood; REML: restricted maximum likelihood; AIC: Akaike information criterion; BIC: Bayesian information criterion; AICc: corrected Akaike information criterion.

Estimation Method	Observations	Log Likelihood	Deviance	AIC	BIC	AICc
ML	19	-34.02	158.95	72.04	73.93	72.79
REML	19	-32.70	65.40	69.40	71.18	70.20

Assessment of Small-Study Effects and Publication Bias

**Figure 3:**
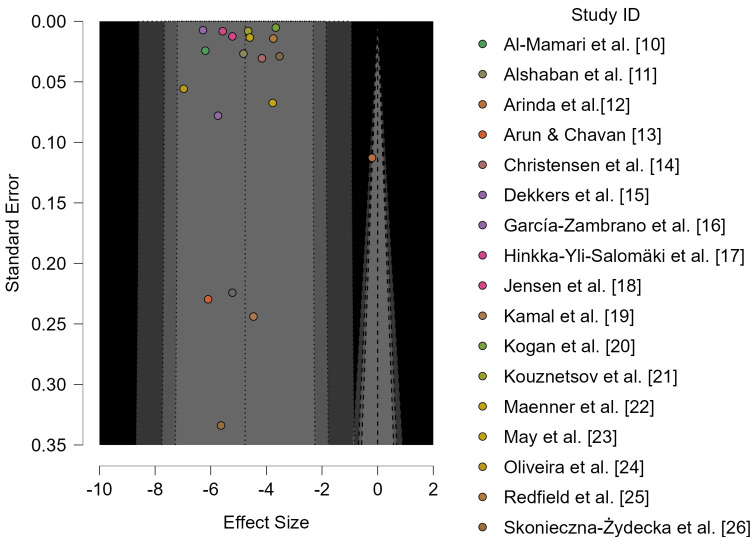
Funnel plot of included studies assessing pooled ASD prevalence The pooled logit transformed prevalence (μ = -4.762; 95% CI: -5.432, -4.092; p < 0.001) corresponds to an estimated prevalence of 0.8% (95% CI: 0.4%-1.7%) on the proportion scale. ASD: autism spectrum disorder.

Funnel plot inspection and formal asymmetry tests were conducted to assess potential small-study effects. Neither the meta-regression test (z = -0.326, p = 0.744) nor the weighted regression test (t = -0.302, df = 17, p = 0.767) indicated statistically significant funnel plot asymmetry. However, given the modest number of studies (n = 19), these tests may have limited power. Accordingly, absence of statistical significance should not be interpreted as definitive evidence of absence of publication bias (Table 5; Figure 3)*.*

**Table 5:** Funnel plot and asymmetry test results for ASD prevalence meta-analysis Estimates μ and τ are on the logit-transformed prevalence scale. Meta-regression and weighted regression tests assess funnel plot asymmetry; non-significant p-values indicate minimal evidence of publication bias. ASD: autism spectrum disorder.

Parameter/Test	Estimate	95% CI Lower	95% CI Upper	Test Statistic	df/z	p
Pooled logit prevalence (μ)	-4.762	-5.432	-4.092	—	—	<0.001
Between-study SD (τ)	1.484	1.118	2.197	—	—	<0.001
Meta-regression asymmetry test	-0.326	-4.669	-3.782	z = -0.326	—	0.744
Weighted regression asymmetry test	-0.302	-4.654	-3.920	t = -0.302	17	0.767

Influence and Sensitivity Analyses

**Figure 4:**
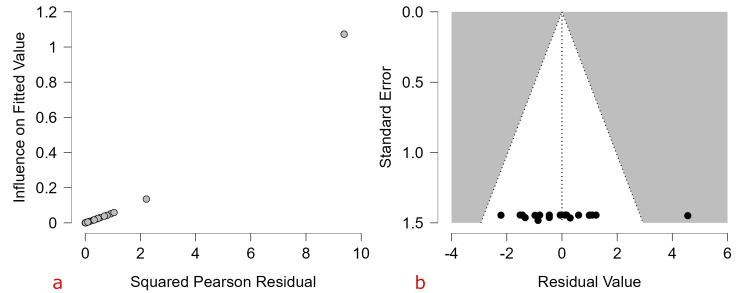
Influence and sensitivity analyses (a) Baujat plot and (b) residual funnel illustrating each study’s contribution to overall heterogeneity (x-axis) and influence on the pooled effect size (y-axis). Most studies cluster near the bottom-left corner, while one studies in the extreme upper-right appear influential and may disproportionately affect the pooled estimate.

A Baujat plot was generated to evaluate each study’s contribution to overall heterogeneity and influence on the pooled estimate. Most studies contributed modestly to both heterogeneity and the overall effect size. One clinic-based study contributed disproportionately to the Q statistic and exerted notable influence on the pooled prevalence estimate. Leave-one-out sensitivity analyses did not materially change the pooled estimate, suggesting that the overall summary effect was not unduly driven by the single influential study [12] (Figure 4).

Subgroup Meta-Analysis

**Figure 5:**
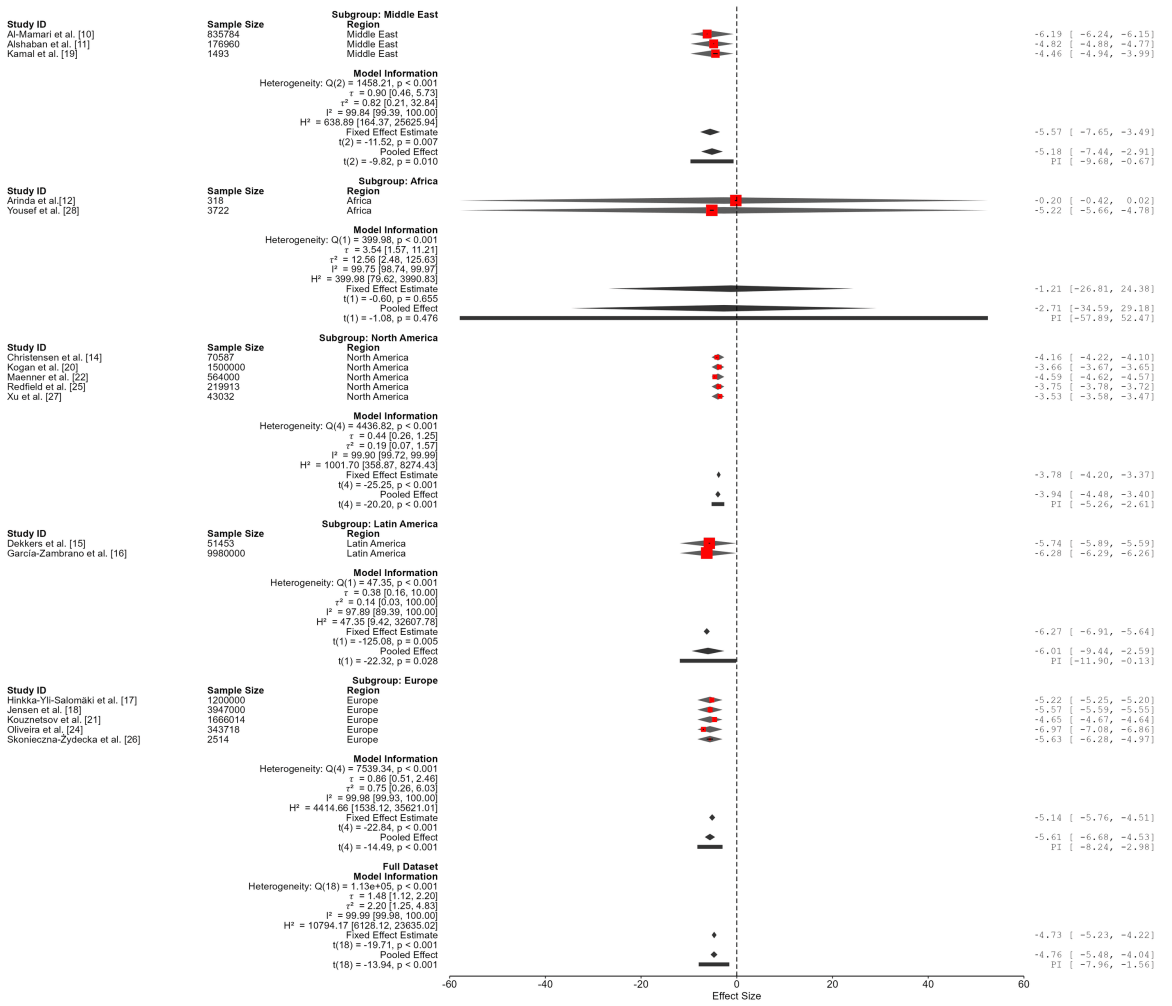
Forest plot of included studies assessing pooled ASD prevalence The pooled logit-transformed prevalence (μ = -4.762; 95% CI: -5.432, -4.092; p < 0.001) corresponds to an estimated prevalence of 0.8% (95% CI: 0.4%-1.7%) on the proportion scale. ASD: autism spectrum disorder.

A subgroup meta-analysis by world region explored potential regional difference in ASD prevalence. Within-region heterogeneity remained extremely high across all regions (I² > 97%), indicating substantial variability even among geographically grouped studies: Middle East, 0.6% (95% CI: 0.06%-5.2%); Africa, 6.3% (95% CI extremely wide due to sparse data); North America, 1.9% (95% CI: 1.1%-3.2%); Latin America, 0.2% (95% CI: 0.01%-7.0%); and Europe, 0.4% (95% CI: 0.1%-1.1%).The African subgroup demonstrated the largest between-study variance (τ² = 12.56), further reflecting instability attributable to the limited number of studies and marked methodological differences in sampling and diagnostic ascertainment. This estimate should not be interpreted as representative of continental prevalence, but rather as an indication of sparse and methodologically diverse data within the region. The test for subgroup differences was statistically significant (Qₘ (4) = 45.37, p < 0.001). However, given the consistently high within-region heterogeneity and small subgroup sizes, particularly for Africa and Latin America, these findings should be interpreted cautiously. The wide confidence and prediction intervals suggest that observed regional differences are likely driven primarily by methodological and contextual variation rather than definitive epidemiological disparities (Table 6; Figure 5).

**Table 6:** Pooled prevalence estimates and heterogeneity by region Subgroup analyses were conducted using a random-effects model. Effect sizes were transformed from log odds to proportions and are reported as percentages. CI: confidence interval; PI: prediction interval; Qₑ: within-subgroup heterogeneity statistic; τ²: between-study variance. All regions showed extremely high heterogeneity (I² > 97%). The African subgroup exhibited the largest τ², reflecting very high uncertainty likely due to the small number of studies and substantial between-study variability. The test for subgroup differences was statistically significant (Qₘ (4) = 45.37, p < 0.001). *Prompts sensitivity analysis.

Region	Pooled prevalence (%)	95% CI (%)	95% PI (%)	Qₑ (df)	p	τ²	I² (%)
Middle East	0.6	0.06-5.2	0.006-33.8	1,458.21 (2)	<0.001	0.82	99.84
Africa*	6.3	~0.0-100.0	~0.0-100.0	399.98 (1)	<0.001	12.56	99.75
North America	1.9	1.1-3.2	0.5-6.8	4,436.82 (4)	<0.001	0.19	99.90
Latin America	0.2	0.01-7.0	0.001-46.8	47.35 (1)	<0.001	0.14	97.89
Europe	0.4	0.1-1.1	0.03-4.8	7,539.34 (4)	<0.001	0.75	99.98

By diagnostic framework, the pooled prevalence estimate varied with DSM-IV studies (n = 5) yielded a pooled prevalence of 2.3% (95% CI: 0.9%-4.3%), whereas ICD-10 studies (n = 6) showed 0.33% (95% CI: 0.14%-0.55%). Combined DSM-IV + ICD-10 studies (n = 3) produced 0.57% (95% CI: 0.23%-1.0%), and DSM-IV + DSM-5 studies (n = 2) yielded 0.20% (95% CI: 0.03%-0.75%). Subgroups with less than two studies were not pooled. Heterogeneity remained very high across all diagnostic subgroups (I² > 99%), indicating persistent methodological and population-level differences. These subgroup estimates should therefore be interpreted as descriptive patterns rather than definitive comparative prevalence rates (Table 7).

**Table 7:** Pooled prevalence estimate of autism spectrum disorder by diagnostic framework and heterogeneity statistics (DSM-IV, DSM-5, ICD-10, and CASI Criteria) Diagnostic frameworks: DSM-IV: Diagnostic and Statistical Manual of Mental Disorders, Fourth Edition (4th edition of the American Psychiatric Association’s manual for diagnosing mental disorders); DSM-5: Diagnostic and Statistical Manual of Mental Disorders, Fifth Edition; ICD-10: International Classification of Diseases, 10th Revision (WHO’s 10th revision disease classification system including mental disorders); CASI: Child and Adolescent Symptom Inventory (standardized rating scale assessing psychiatric symptoms in children and adolescents).

Diagnostic Framework	n studies	Pooled Prevalence (%)	95% CI	τ²	I² (%)
DSM-IV	5	2.3	0.9-4.3	4.661	99.91
ICD-10	6	0.33	0.14-0.55	0.672	99.98
DSM-IV + ICD-10	3	0.57	0.23-1.0	0.818	99.84
DSM-IV + DSM-5	2	0.20	0.03-0.75	0.123	99.61
DSM-5	<2	—	—	—	—
DSM-IV/CASI	<2	—	—	—	—
ICD-10/DSM-5	<2	—	—	—	—

Exploratory Meta-Regression Analysis

Univariate meta-regression analyses were conducted to explore potential study-level moderators of ASD prevalence. Predictors examined individually included mean age, diagnostic framework, study setting, and geographic region. Most predictors apart from study setting (F (6,12) = 15.16, p < 0.001, R² = 82.8%) did not reach statistical significance including mean age (F (1,12) = 1.83; p = 0.202; β = 0.46; 95% CI: -0.28, 1.19; R² = 6%), diagnostic framework (F (6,12) = 1.35, p = 0.309, R² = 10.6%) and geographic region (F (6,12) = 2.44, p = 0.089), indicating that sampling context explained a substantial proportion of between-study heterogeneity (Table 8).

**Table 8:** Univariate meta-regression screening of moderators β: regression coefficient; R²: proportion of between-study heterogeneity explained; MVRA: multivariable meta-regression analysis.

Predictor	F (df₁, df₂)	p	β	95% CI	R² (%)	Retained for MVRA
Mean age	1.83 (1,12)	0.202	0.46	-0.28 to 1.19	6	No
Diagnostic framework	1.35 (6,12)	0.309	—	—	10.6	Yes
Study setting	15.16 (6,12)	<0.001	—	—	82.8	Yes
Geographic region	2.44 (6,12)	0.089	—	—	—	Yes

Reduced Meta-Regression Analysis

**Figure 6:**
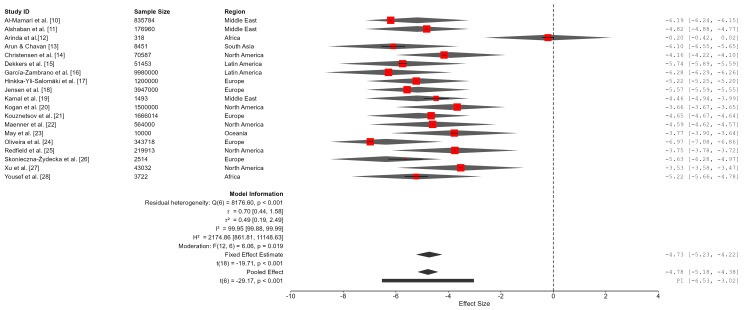
A forest plot of reduced meta-regression analysis

A reduced multivariable meta-regression model was conducted including diagnostic framework, study setting, and geographic region as predictors, with sparse categories collapsed to ensure model stability. The model was statistically significant (Fₘ (12,6) = 6.06, p = 0.019) and explained 77.95% of the between-study variance. Diagnostic framework (F (6,6) = 6.14, p = 0.022), study setting (F (2,6) = 13.67, p = 0.006), and geographic region (F (4,6) = 8.91, p = 0.011) were significant moderators of ASD prevalence. Specifically, use of combined DSM-IV and ICD-10 criteria was associated with lower prevalence estimates (b = -5.36; 95% CI: -8.91, -1.81; p = 0.010). Population-based (b = 6.24; 95% CI: 3.25, 9.23; p = 0.002) and institution-based settings (b = 5.02; 95% CI: 2.51, 7.53; p = 0.003) were associated with higher prevalence relative to the reference category. Regionally, studies from Europe (b = -4.41, p = 0.014), Latin America (b = -5.54, p = 0.001), North America (b = -4.77, p = 0.007), and Oceania (b = -4.79, p = 0.007) reported significantly lower prevalence compared with the reference region. Although the model accounted for a substantial proportion of between-study heterogeneity (R² = 77.95%), residual heterogeneity remained very high (I² = 99.95%), indicating persistent unexplained variability across study populations and methodological characteristics (Table 9; Figure 6).

**Table 9:** Reduced meta-regression screening of moderators Reference categories: Diagnostic Criteria = DSM-IV baseline; Setting = Community-based; Region = Middle East/South Asia (removed due to sparse data); Effect sizes transformed from log odds to proportions; Knapp-Hartung adjustment applied; Residual heterogeneity remains high (I² = 99.95%), indicating considerable variability across studies. Diagnostic frameworks: DSM-IV: Diagnostic and Statistical Manual of Mental Disorders, Fourth Edition; DSM-5: Diagnostic and Statistical Manual of Mental Disorders, Fifth Edition; ICD-10: International Classification of Diseases, 10th Revision; CASI: Child and Adolescent Symptom Inventory.

Moderator	Category	Estimate (b)	95% CI	p-value
Diagnostic criteria	DSM-IV	0.85	-1.59, 3.28	0.428
	DSM-IV, CASI	-0.03	-3.56, 3.50	0.985
	DSM-IV, DSM-5	0.68	-1.42, 2.79	0.458
	DSM-IV, ICD-10	-5.36	-8.91, -1.81	0.010
	ICD-10	-0.91	-4.72, 2.89	0.579
	ICD-10, DSM-5	1.07	-1.37, 3.50	0.324
Setting	Population-based	6.24	3.25, 9.23	0.002
	Institution-based	5.02	2.51, 7.53	0.003
Region	Europe	-4.41	-7.57, -1.25	0.014
	Latin America	-5.54	-7.99, -3.08	0.001
	North America	-4.77	-7.70, -1.83	0.007
	Oceania	-4.79	-7.73, -1.85	0.007

Diagnostic Analysis on Reduced Meta-Regression

**Figure 7:**
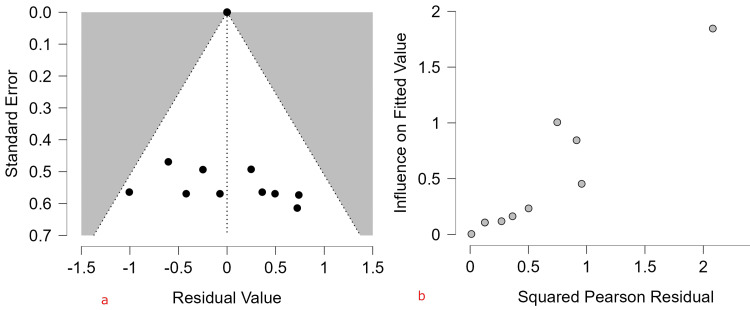
(a) Residual funnel plot (n = 11) and (b) influence diagnostics (n = 9) from the reduced multivariable meta-regression model of ASD prevalence ASD: autism spectrum disorder.

Residuals plotted against standard error showed most studies clustering symmetrically within the funnel, although residual heterogeneity remained high (I² = 99.95%). Influence diagnostics revealed that, while most studies exert modest influence, one observation displayed both high residual and high influence, indicating disproportionate impact on model fit despite the model explaining 77.95% of between-study variance (Figure 7).

Bayesian Analysis

To complement the frequentist meta-analysis, we conducted a Bayesian random-effects meta-analysis using weakly informative priors (Normal (0,1) for pooled logit prevalence; Half-Cauchy (0,1) for between-study heterogeneity, τ). This approach accounts for extreme heterogeneity (I² ≈ 100%) and the modest number of studies, providing full posterior distributions and credible intervals. The posterior mean logit prevalence was -4.15 (95% credible interval (CrI): -4.89, -3.19), corresponding to 1.55% prevalence (95% CrI: 0.75%-4.1%), almost double the frequentist estimate of 0.8% (95% CI: 0.4%-1.7%). Posterior heterogeneity (τ = 1.66; 95% CrI: 1.16-2.48), and the predictive interval (0.05%-36%) reflects substantial variability across populations. These results confirm the robustness of our findings, highlighting that observed variability is primarily driven by study- and population-level differences (Table 10).

**Table 10:** Comparison of frequentist and Bayesian random-effects meta-analysis of ASD prevalence estimate This table summarizes and compares results from frequentist and Bayesian random-effects meta-analyses of autism spectrum disorder (ASD) prevalence across 19 studies; the Bayesian model applied weakly informative priors (Normal (0,1) for pooled logit prevalence and Half-Cauchy (0,1) for heterogeneity, τ) to address extreme heterogeneity (I² ≈ 100%) and the modest study count, producing posterior distributions and 95% credible interval (CrI); the frequentist approach reports standard pooled estimates with 95% confidence interval (CI); reported metrics include pooled logit prevalence, back-transformed prevalence, heterogeneity (τ and τ²), I², prediction intervals, and Bayesian convergence diagnostics; overall, Bayesian estimates align closely with frequentist results while providing a more comprehensive probabilistic characterization of uncertainty. ESS: Effective Sample Size.

Metric	Frequentist	Bayesian
Pooled logit prevalence (μ)	-4.762	-4.147
Pooled prevalence (%)	0.8% (95% CI: 0.4%-1.7%)	1.55% (95% CrI: 0.75%-4.1%)
Heterogeneity (τ)	1.484	1.659
Heterogeneity variance (τ²)	2.20	2.871
I²	99.99%	99.99%
Prediction interval	0.03%-17.3%	0.05%-36%
Convergence diagnostics	—	R-hat ≤ 1.001, ESS > 3800

Bayesian Model Diagnostics

**Figure 8:**
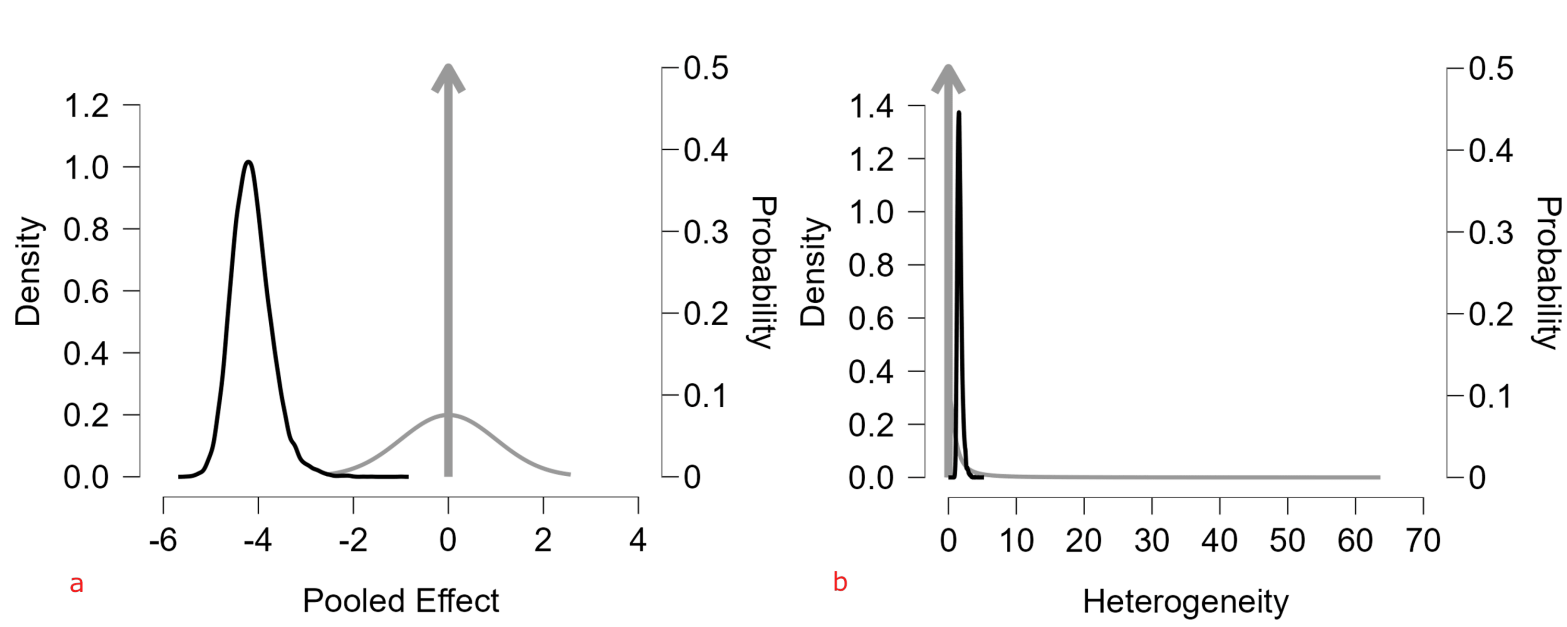
Prior and posterior distributions for the (a) pooled ASD prevalence (logit scale) and (b) heterogeneity parameter τ Priors reflect weakly informative assumptions; posteriors indicate data-driven refinement of parameter estimates with credible intervals. ASD: autism spectrum disorder.

The prior and posterior distributions of the pooled prevalence (logit scale) and heterogeneity parameter τ are shown. The posterior distribution is notably narrower than the prior, indicating that the observed data provided substantial information to refine parameter estimates. The pooled logit prevalence had a posterior mean of -4.15 (95% credible interval: -4.89, -3.19), corresponding to an estimated prevalence of approximately 1.55% on the proportion scale after back-transformation. The heterogeneity parameter τ also showed a posterior distribution consistent with substantial between-study variance (Figure 8).

Markov Chain Monte Carlo (MCMC) Trace Plots and Convergence

**Figure 9:**
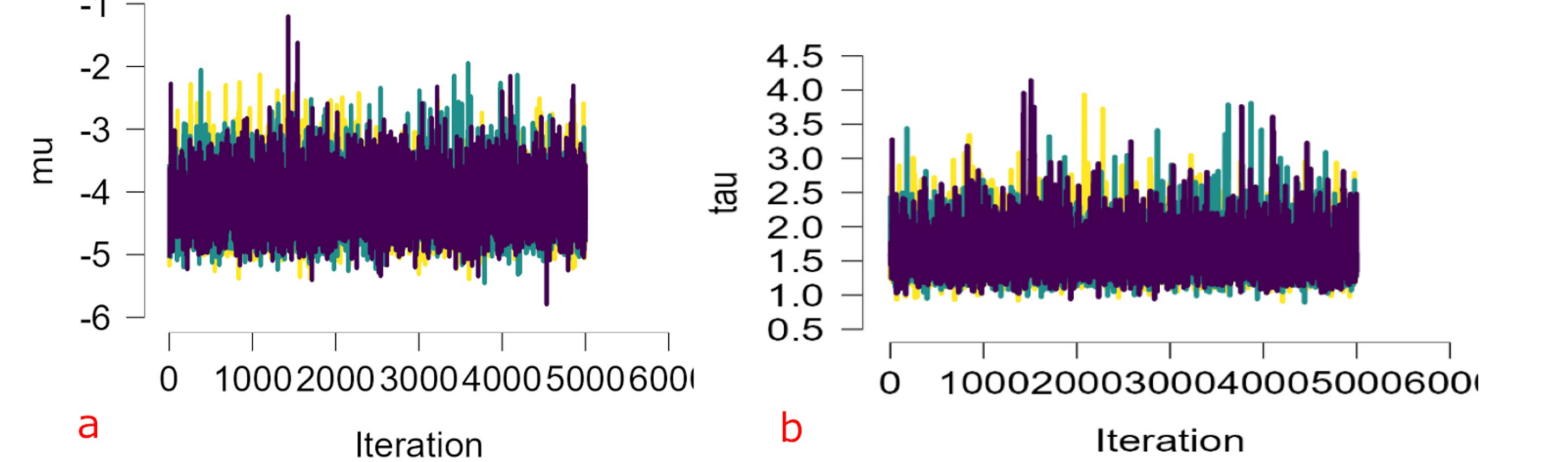
Markov Chain Monte Carlo (MCMC) trace plots for the (a) pooled logit prevalence and (b) heterogeneity parameter τ across multiple chains The plots show good mixing and convergence, evidenced by stable traces without trends or drift.

Trace plots for the pooled effect and heterogeneity parameter (τ) demonstrated good mixing and stationarity across chains, with no evident trends or pathological autocorrelation. Convergence diagnostics indicated satisfactory model performance: R-hat values were approximately 1.00 for all parameters (μ = 1.000; τ = 1.001), and effective sample sizes exceeded 3,000, supporting adequate convergence and sampling efficiency (Figure 9).

Bayesian Model Sensitivity Analysis (Clinic-Based Study Removed)

**Figure 10:**
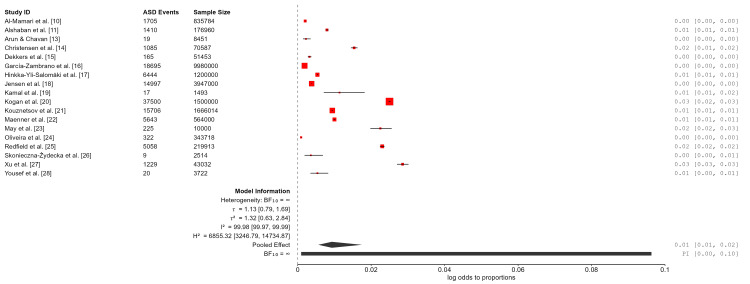
Forest plot of Bayesian sensitivity analysis excluding the extreme outlier study Extreme outlier study (Arinda et al. [12]). ASD: autism spectrum disorder.

A Bayesian sensitivity analysis was conducted to assess the influence of Arinda et al. study on the pooled ASD prevalence [12]. Using the same model priors (Normal (0,1) for pooled effect and Half-Cauchy (0,1) for heterogeneity), and excluding the outlier, the posterior mean prevalence was 0.9% (95% CrI: 0.6%-1.8%), with substantial between-study heterogeneity (τ = 1.14, I² = 99.98%). The posterior estimate was nearly identical to the frequentist pooled prevalence of 0.8% (95% CI: 0.4%-1.7%), indicating that the overall pooled estimate is robust and not unduly influenced by the extreme study. The corresponding prediction interval (0.09%-9.5%) also remained wide, reflecting high variability across populations (Table 11; Figure 10).

**Table 11:** Comparison table (Bayesian vs frequentist) Bayesian estimates use weakly informative priors; sensitivity analysis shows that excluding the extreme study has minimal effect on the pooled prevalence; τ = between-study SD on logit scale; I² quantifies proportion of variance due to heterogeneity. CrI: credible interval.

Model/Sensitivity	Pooled Prevalence	95% Interval	Heterogeneity (τ)	I²
Frequentist (all studies)	0.8%	0.4%-1.7%	1.48	99.99%
Bayesian (all studies)	1.5%	0.75%-4.1%	1.63	99.99%
Bayesian (sensitivity, outlier clinic-based study removed)	0.9%	0.6%-1.8%	1.14	99.98%

Discussions

Principal Findings

This meta-analysis synthesized 19 studies published between 2004 and 2025 to examine reported global prevalence of autism spectrum disorder (ASD) and to identify drivers of between-study heterogeneity. The pooled prevalence was 0.8% (95% CI: 0.4%-1.7%) (one in 125) individuals meeting diagnostic criteria within the populations studied. However, prevalence varied dramatically across contexts; the 0.8% estimate represents an average across highly heterogeneous surveillance systems rather than a single globally representative rate.

Between-study heterogeneity was high (I² ≈ 100%), and the 95% prediction interval (0.03%-17.3%) indicated that prevalence estimates in comparable future populations may differ substantially depending on diagnostic framework, study setting, and regional surveillance capacity. Although heterogeneity was substantial, pooling was retained because the objective was not to derive a single universal prevalence rate, but to quantify both the magnitude of variation and the study-level factors contributing to that variability across global contexts. Methodological guidance supports the use of random-effects meta-analysis under conditions of marked heterogeneity when accompanied by prediction intervals and moderator analyses that explicitly model between-study variance. Accordingly, the pooled estimate should be interpreted as a descriptive statistical summary across diverse settings rather than a precise global benchmark. The magnitude of heterogeneity likely reflects differences in case ascertainment strategies, diagnostic frameworks (DSM versus ICD systems), sampling approaches, healthcare infrastructure, and sociocultural recognition of developmental conditions. Thus, variability itself, rather than the pooled point estimate, emerges as the central finding of this synthesis.

Regional Variation

Subgroup analyses by world region revealed pooled prevalence estimates of 0.6% for the Middle East, 6.3% for Africa, 1.9% for North America, 0.2% for Latin America, and 0.4% for Europe. Within-region heterogeneity remained high (I² > 97%), and confidence intervals were particularly wide for Africa and Latin America. The African estimate (6.3%) should not be interpreted as a definitive continental prevalence rate, but rather as a reflection of sparse data, limited diagnostic infrastructure, and substantial methodological variability across studies. Systematic reviews of ASD research in sub-Saharan Africa have similarly highlighted small sample sizes, clinic-based recruitment, and inconsistent diagnostic tools as major limitations [30,31]. Similar challenges have been reported in other underrepresented regions. Recent evidence from the Middle East and North Africa demonstrates substantial variability in ASD prevalence estimates, largely driven by differences in diagnostic methods and study design [32]. In Latin America and other low- and middle-income regions, global burden estimates also highlight disparities in surveillance systems, awareness, and diagnostic capacity as key contributors to inconsistent prevalence estimates [33]. The wide prediction intervals observed across all regions further indicate that regional differences may be driven more by methodological and surveillance-related factors.

Methodological Moderators

Univariable meta-regression analyses identified study setting as the only statistically significant moderator, whereas mean age, diagnostic framework, and geographic region were not independently associated with prevalence. In multivariable models including diagnostic framework, study setting, and region, all three variables emerged as significant predictors and collectively explained approximately 78% of between-study variance (R² = 77.95%). Consistent with our a priori hypothesis, methodological characteristics, including diagnostic framework and study setting, accounted for substantially more between-study variability than demographic factors such as mean age.

These findings support the premise that differences in case ascertainment and surveillance practices, rather than population composition alone, are primary drivers of observed prevalence variation. However, given the modest number of included studies (n = 19) and limited residual degrees of freedom, these multivariable findings should be interpreted cautiously due to potential overfitting. Importantly, residual heterogeneity remained high (I² = 99.95%), indicating that substantial unexplained variability persists despite accounting for key methodological characteristics. Contrary to an earlier report suggesting age-related differences in ASD prevalence [34], mean age was not a statistically significant moderator in univariable analyses in the present study. This suggests that methodological and contextual factors may exert stronger influence on reported prevalence than age distribution alone.

Collectively, these findings reinforce prior evidence that prevalence estimates are highly sensitive to study design, case-finding strategies, and diagnostic criteria [32,33]. Structured diagnostic tools and clinic-based sampling frames may elevate case detection relative to community-based or registry approaches, contributing to observed variability. 

Sensitivity and Robustness

Sensitivity analyses demonstrated that Arinda et al. study exerted disproportionate influence on heterogeneity statistics and pooled estimates [12]. Excluding this study modestly altered the pooled prevalence but did not materially change overall conclusions. Both frequentist and Bayesian sensitivity analyses yielded comparable pooled estimates following exclusion of the influential study, reinforcing the relative stability of the central estimate despite persistent heterogeneity. These findings underscore the importance of influence diagnostics in ASD meta-analyses, where extreme values can meaningfully affect summary estimates [35,36].

Bayesian Interpretation

To complement frequentist modeling, a Bayesian random-effects meta-analysis was conducted using weakly informative priors. The Bayesian pooled prevalence estimate (1.55%; 95% CrI: 0.75%-4.1%) was moderately higher than the frequentist estimate (0.8%), likely reflecting partial shrinkage toward the prior mean under extreme heterogeneity. Posterior heterogeneity remained substantial (τ = 1.66), and the predictive interval (0.05%-36%) was extremely wide. Importantly, Bayesian modeling did not materially reduce heterogeneity; rather, it provided full posterior and predictive distributions that reinforced the central conclusion that ASD prevalence varies dramatically across contexts. Convergence diagnostics (R-hat ≈ 1.00; large effective sample sizes) supported computational stability, increasing confidence in the robustness of posterior inference.

Comparison With Prior Literature

Large-scale global reviews have generally reported ASD prevalence between 0.4% and 1.0% [35,37], aligning with the lower bound of the present pooled estimate (0.8%). More recent surveillance data from high-income countries, particularly North America, report higher prevalence estimates consistent with the elevated regional estimate observed here (1.9%) [20,22]. Importantly, many earlier global syntheses relied on data collected prior to major expansions in ASD surveillance systems, refinements in diagnostic criteria, and increased public and professional awareness. The present analysis, spanning 2004-2025, captures a period marked by substantial growth in structured screening, registry-based monitoring, and broader diagnostic inclusion under DSM-IV and DSM-5 frameworks. As such, differences between earlier pooled estimates and more recent regional figures likely reflect evolving case ascertainment practices rather than true shifts in underlying population incidence.

Multi-country analyses have consistently demonstrated that variability in ASD prevalence is strongly influenced by surveillance infrastructure, availability of trained diagnostic providers, and the use of differing diagnostic criteria [38,39]. The present findings extend this literature by quantifying the extent to which diagnostic framework, study setting, and geographic region explain between-study variance (R² = 78%) in multivariable models, while also demonstrating that substantial residual heterogeneity persists. This pattern reinforces prior conclusions that methodological differences, including structured assessment tools (Autism Diagnostic Interview-Revised (ADI-R), Childhood Autism Rating Scale (CARS)), clinic-based recruitment, and registry linkage, tend to produce higher prevalence estimates than interview-only or community-based approaches [26].

Several studies have documented pronounced geographic disparities in ASD prevalence, particularly in low-resource settings. Systematic reviews of ASD research in sub-Saharan Africa emphasize the scarcity of population-based data, reliance on clinic or convenience samples, and inconsistent use of standardized diagnostic instruments [30,31]. Reported prevalence estimates in African settings vary widely, ranging from community-based estimates below 1% to substantially higher clinic-based figures [40]. The wide confidence and prediction intervals observed in the African subgroup mirror prior reviews documenting sparse population-based data and inconsistent diagnostic practices in sub-Saharan Africa. Rather than indicating true continental prevalence, these findings highlight ongoing gaps in surveillance capacity and epidemiologic infrastructure.

In contrast to earlier evidence suggesting age-related increases in ASD [34], mean age was not a statistically significant moderator in univariable analyses in the present study. This divergence may reflect the stronger influence of methodological characteristics relative to demographic composition in shaping reported prevalence across studies. While prior work has linked broader screening and older age cohorts to higher observed prevalence [36,41], the current findings suggest that diagnostic framework and study context may exert greater explanatory power in contemporary datasets. The sensitivity of meta-regression results to the single influential study (Arinda et al. [12]) also aligns with prior observations that ASD prevalence syntheses are particularly susceptible to outliers due to methodological diversity [35]. The consistency of findings across frequentist and Bayesian sensitivity analyses in the present study strengthens confidence that, despite extreme heterogeneity, the overall pattern of variability is robust.

These patterns, including substantial between-study heterogeneity, broad prediction intervals, and variation linked to methodological and regional differences, are consistent with prior global meta-analytic evidence showing that high heterogeneity and context-dependent variation are typical in pooled analyses across diverse study settings [42,43]. The degree of heterogeneity identified here aligns with prior Bayesian evidence syntheses, where hierarchical models routinely reveal substantial between-study variability and correspondingly wide prediction intervals when pooling globally diverse data [44-47]. Collectively, these findings reinforce that global ASD prevalence estimates are shaped more by methodological and health system factors than by intrinsic cross-national epidemiologic differences [32,48-51].

Strengths and Limitations

This study has several strengths. It synthesizes recent global data (2004-2025), applies both frequentist and Bayesian random-effects models, reports prediction intervals, and systematically evaluates moderators and influence diagnostics. The inclusion of Bayesian modeling represents a methodological strength, allowing probabilistic interpretation under conditions of extreme heterogeneity. Nonetheless, limitations warrant consideration. The multivariable meta-regression included multiple categorical predictors relative to the modest number of studies (n = 19), which may increase the risk of overfitting and unstable parameter estimates. Accordingly, moderator findings should be interpreted as exploratory and hypothesis-generating rather than confirmatory. The exceptionally high heterogeneity limited the interpretability of a single pooled prevalence as a definitive global benchmark. Given such variability, the pooled estimate should be viewed as a descriptive average rather than a precise global benchmark. Residual heterogeneity remained substantial even after multivariable adjustment, indicating that unmeasured methodological or contextual factors likely contribute to observed differences. Additionally, the multivariable meta-regression included several predictors relative to the modest number of studies, raising potential concerns about model stability despite statistical significance. The restriction to English-language publications may have excluded relevant data from non-English-speaking regions, potentially introducing language bias and affecting regional representation. Limited data from Africa and Latin America further constrained precision in those subgroups.

Implications and Future Directions

From a global public health perspective, accurate prevalence estimation is essential for service planning, resource allocation, and early intervention policy development. The findings highlight the urgent need for standardized case ascertainment strategies, harmonized diagnostic frameworks, and improved surveillance infrastructure, particularly in low-resource settings. Expanding the use of validated diagnostic tools and strengthening national registries may improve comparability across countries. Future research should prioritize population-based surveillance in underrepresented regions, transparent reporting of diagnostic criteria and sampling strategies, and longitudinal designs capable of disentangling changes in detection from true epidemiological trends.

## Conclusions

This synthesis demonstrates that reported ASD prevalence varies widely across global contexts, reflecting substantial differences in surveillance systems and study methodologies. Variations in diagnostic frameworks, study settings, and geographic regions account for a considerable portion of the observed heterogeneity, although notable variability remains. These findings indicate that global prevalence cannot be adequately represented by a single universal estimate; rather, reported rates reflect complex interactions among diagnostic practices, surveillance intensity, and sociocultural context. Future efforts should prioritize harmonized methodologies, expanded population-based surveillance in underrepresented regions, and transparent reporting of diagnostic and sampling procedures to enhance comparability and better inform public health planning.

## Appendices

Supplementary material 1

**Table 12:** Breakdown of excluded studies (n = 635) This table provides a detailed breakdown of the 635 full-text articles excluded at the screening stage by reason (non-English publications, case reports, case series, reviews, meta-analyses, editorials, and commentaries).

Reason for Exclusion	Number of Studies (n)
Non-English publications	85
Case reports	70
Case series	75
Reviews	160
Meta-analyses	90
Editorials	80
Commentaries	75
Total	635

Supplementary material 2

**Table 13:** Database-specific search strategies and records retrieved Total records retrieved: 1,040. This table summarizes the search strategies used in each database, along with records retrieved, filters/limits applied, and the search date. ASD: autism spectrum disorder.

Database	Records Retrieved	Search Strategy	Filters/Limits	Search Date
PubMed/ MEDLINE	415	("Autism Spectrum Disorder"[MeSH] OR "autism spectrum disorder" OR ASD OR autism) AND ("Prevalence"[MeSH] OR prevalence OR epidemiology OR occurrence OR frequency) AND ("Epidemiologic Studies"[MeSH] OR "cross-sectional studies" OR "population-based" OR survey)	English; Humans; 2004-2025	December 31, 2025
Embase	234	('autism spectrum disorder'/exp OR 'autism spectrum disorder' OR ASD OR autism) AND ('prevalence'/exp OR prevalence OR epidemiology OR occurrence OR frequency) AND ('cross-sectional study'/exp OR 'population-based' OR survey)	English; Humans; 2004-2025	December 31, 2025
Web of Science	257	TS=("autism spectrum disorder" OR autism OR ASD) AND TS=(prevalence OR epidemiology OR occurrence OR frequency) AND TS=("cross-sectional" OR "population-based" OR survey)	English; 2004-2025	December 31, 2025
Scopus	134	TITLE-ABS-KEY("autism spectrum disorder" OR autism OR ASD) AND TITLE-ABS-KEY(prevalence OR epidemiology OR occurrence OR frequency) AND TITLE-ABS-KEY("cross-sectional" OR "population-based" OR survey)	English; 2004-2025	December 31, 2025
